# Our European Correspondence

**Published:** 1867-10

**Authors:** 


### OUR EUROPEAN CORRESPONDENCE.

We publish in another column a second letter from our distinguished friend, L. P. Yandell, M. D. His letters are very able, and show an immense amount of laborious enquiry in all matters appertaining to the profession of medicine. He has recently gone to Paris, from which place we hope to hear from him, in time for our next monthly issue.

